# Constructing gene network for type 1 narcolepsy based on genome-wide association study and differential gene expression analysis (STROBE)

**DOI:** 10.1097/MD.0000000000019985

**Published:** 2020-05-01

**Authors:** Hui Ouyang, Shiying Wang, Qiwen Zheng, Jun Zhang

**Affiliations:** a Department of Clinical Neurology, Peking University, People's Hospital; b Department of Epidemiology and Biostatistics, Peking University Health Science Center, Beijing, China.

**Keywords:** gene, gene network, neurodegeneration, pathway, single nucleotide polymorphism

## Abstract

Although many genes that affect narcolepsy risk have been identified, the interactions among these genes are still unclear. Moreover, there is a lack of research on the construction of the genetic network of narcolepsy. To screen candidate genes related to the onset of narcolepsy type 1, the function and distribution of important genes related to narcolepsy type 1 were studied and a gene network was constructed to study the pathogenesis of narcolepsy type 1.

A case-control study (observational study) of 1075 Chinese narcoleptic patients and 1997 controls was conducted. The gene-sequencing data was analyzed using genome-wide association analysis. The candidate genes related to narcolepsy were identified by differential gene expression analysis and literature research. Then, the 28 candidate genes were input into the KEGG database and 32 pathway data related to candidate genes were obtained. A gene network, with the pathways as links and the genes as nodes, was constructed. According to our results, *TNF*, *MHC II*, *NFATC2,* and *CXCL8* were the top genes in the gene network.

*TNF*, *MHC II, NFATC2,* and *CXCL8* are closely related to narcolepsy type I and require further study. By analyzing the pathways of disease-related genes and the network of gene interaction, we can provide an outlinefor the study of specific mechanisms of and treatments for narcolepsy.

## 1 Introduction

### 1.1 The epidemiology and pathogenesis of narcolepsy

Narcolepsy is a life-long neurological disorder characterized by excessive daytime sleepiness (EDS), cataplexy, sleep paralysis, hallucinations, and disrupted nocturnal sleep.^[1]^ Epidemiological data show that the global incidence of narcolepsy is 0.03%.^[2]^ Narcolepsy displays a strong genetic predisposition: the incidence of narcolepsy among first-degree relatives of narcolepsy patients is 1% to 2%, 10 to 40 times that of the normal population,^[3]^ and 25% to 31% of identical twins are co-infected.^[4]^

Several studies have found that the cerebrospinal fluid hypothalamic secretion, which plays an important role in promoting awakening,^[5]^ is lower in narcolepsy patients.^[6]^ Autopsy results have shown that 90% to 95% of hypothalamic-secretin-producing neurons were lost in some patients with narcolepsy, but the mechanism of neuron loss is still unclear. Recently, the genetic risk of narcolepsy is evaluated based on the carriage of *HLA-DQB1*^*∗*^*06:02*, an important but imperfect predictor of narcolepsy,^[7,8]^ since 10% to 40% of individuals in the unaffected population carry it as well.^[9]^ The correlation between influenza A (H1NI) epidemics and increased incidence of narcolepsy suggest that influenza-virus-induced autoimmunity is the possible route of pathogenesis for narcolepsy.^[10]^ However, no specific autoantibodies or T cells have been found to cross-react with hypocretin neurons,^[11]^ hence, the mechanisms of disease occurrence and development still need further study.

### 1.2 The Genes associated with narcolepsy

Gene-related studies of narcolepsy mainly use genome-wide association analysis and other pathological analyses. Genome-wide association study (GWAS) is a method to identify disease or trait-related loci through genetic variation, mainly single nucleotide polymorphism (SNP), using the linkage disequilibrium principle. Many genes have been identified as narcolepsy risk factors in international SNP-based GWASs of narcolepsy type I across different ethnic groups. A Japanese team found that the *CPT1B* gene (carnitine palmitoyltransferase 1B) and the *CHKB* gene (choline kinase B), both involved in REM (rapid eye movement) regulation, are associated with narcolepsy type I.^[12]^ Subsequent genome-wide association analysis revealed that narcolepsy is highly associated with the *TCR alpha* (T cell receptor alpha chain) loci,^[13]^ which has been repeated in subsequent studies.^[14]^ The *TCR alpha* locus produces a unique protein in T lymphocytes and plays an important role in identifying antigens bound to HLA. A GWAS study in a Chinese population showed that *PR2Y11, PPAN, TRB, IL10RB,* and *ZN365* may predict susceptibility to narcolepsy.^[15]^ Among them, *P2RY11* is highly expressed in cytotoxic T lymphocytes and plays a role in regulating cell migration, cytokine release, and apoptosis.^[16]^ Potential susceptibility genes identified by genome-wide association analysis include *CTSH, TNFSF4,*^[17]^*CCR1, CCR3,*^[18]^*CLOCK,*^[19]^*TEAD4, UBXN2B,*^[20]^*EIF3G.*^[21]^ In addition, studies combining GWAS with gene pathway information suggest that the genes *CACNA1C, NFATC2, POLE, FAM3D,* and *SCP2* may be associated with narcolepsy.^[22]^

Mutant gene research found that *MOG*^[23]^ and *DNMT 1*^[24]^ are associated with narcolepsy. *DNMT1* is expressed in immune cells and plays a role in the differentiation of CD4 + T cells into regulatory T cells. Pathological studies have compared the concentration of molecules in cerebrospinal fluid and blood. In addition to the level of hypocretin (HCRT) mentioned above, the concentrations of *BDNF,*^[25]^ GFAP,^[26]^*TNF alpha,* and *CXCL8*^[27]^ were also significantly different. Differential gene expression studies have found that *IGFBP3* is associated with narcolepsy.^[28]^ However, although so many genetic risk factors for narcolepsy have been identified, the interactions among these genes are still unclear. Moreover, there is a lack of research on the construction of the genetic network of narcolepsy.

## 2 Materials and methods

### 2.1 Ethical approval

The research protocols were approved by the Institutional Review Board Panels on Medical Human Subjects at the Peking University People's Hospital. The control group consisted of 1997 staff members and students from several universities.

#### 2.1.1 Genome-wide association study

##### 2.1.1.1 Study population

The study was conducted on 1,075 patients with narcolepsy type 1. The patients were recruited from the sleep laboratory at Peking University People's Hospital. The narcolepsy patients were diagnosed according to the ICSD-3 diagnostic criteria. Cases were all Chinese, and most of them were of Han descent (95%). Clinical data included the presence or absence of cataplexy, sleepiness, sleep paralysis, hypnogogic hallucination, and disturbed nocturnal sleep. Trained interviewers used structured questionnaires to collect information on demographic variables, medical history, and medications. Informed consent (in accordance with governing institutions) was obtained from all subjects.

##### 2.1.1.2 Quality control of the sample and data filtering

Quality control was conducted according to information from the 1075 narcolepsy patients. We excluded patients with narcolepsy type 2 and patients that were suspected to have errors in their input information. In total, 992 patients passed quality control and continued to the next phase of the study.

##### 2.1.1.3 Genotyping and quality control

DNA samples were genotyped on the Affymetrix Axiom CHB array. Genotypes were called using the Affymetrix Genotyping Console. Imputation of 1000 Genomes project SNPs from this array using IMPUTE2 and the Phase I v2, cosmopolitan (integrated) reference panel, build 37.^[29]^ Individuals with call rate <99%, MAF (Minor Allele Frequency) <1%, HWE (Hardy-Weinberg equilibrium), *P* value < .001, or related were removed, leaving 903 cases and 1997 controls.

##### 2.1.1.4 Population stratification analysis

Principal component analysis of the SNPs within linkage equilibrium (r^2^ < 0.25) was conducted using the PLINK software. Before obtaining the first 20 principal components, people in the cohort who did not cluster with their specified ancestral group (±6SD from the cluster mean on the first 2 principal components) were excluded. To further eliminate the possible impact of population stratification on the results of the association analysis, illness was used as a binary dependent variable to carry out logistic regression and find the principal components with significant differences between the disease group and the control group according to the methods described in the study by Price et al.^[30]^ In subsequent association studies, these principal components were included in the regression analysis as covariates to eliminate them, in order to eliminate the impact of population stratification.

##### 2.1.1.5 Statistical analysis

We conducted the GWAS using the case-control study. Logistic regression was performed using PLINK software. Presence of narcolepsy was the dependent variable. Covariates included the sex of the individual and the first 3 principal components of the principal component analysis. The independent variable was the genotype of the SNP locus. In this study, the error false discovery rate method was used,^[31]^ hence the genome-wide significant *P* value threshold is *P* = 4.2^∗^10^−5^. Genome-wide association studies were performed using regression models using the R software. A Manhattan map and quantile-quantile plot were drawn, and the genomic inflation factor (GIF) was found.^[32]^ For the region containing multiple positive loci, a conditional regression analysis was conducted using the locus with the lowest *P* value to determine the number of loci associated with disease in the region. A map of the region showing genes and degrees of recombination was drawn using the LocusZoom.^[33]^

#### 2.1.2 Expression analysis of differential genes

##### 2.1.2.1 Data source

The data were downloaded from the gene expression omnibus (GEO), and the gene expression data were collected from the genome-wide gene expression profile of the human narcolepsy project. The project used chips to collect gene expression data from the circulating lymphocyte mononuclear cells of 10 white narcolepsy patients matched by age and sex and 10 white, healthy controls.

##### 2.1.2.2 Data processing and Statistical Analysis

The background correction, standardization, and logarithmic transformation of gene expression data were completed according to the original research of Akintomide et al.^[34]^ The gene expression data from the circulating lymphocyte monocytes in the case group and control group were analyzed by ANOVA, and significant differences in gene expression between the 2 groups were compared with the threshold set at *P* < 0.01. The R software was used to cluster the samples and the significant difference genes, and the thermal map was drawn.

#### 2.1.3 Construction of gene network

##### 2.1.3.1 KEGG database

The Kyoto Encyclopedia of Genes and Genomes (KEGG) database was established in 1995 by Kanehisa Laboratory of the Bioinformatics Center of Kyoto University, Japan.^[35]^ The database provides basic information on genes, DNA sequence, chromosome location sequence, chromosome location, and more, as well as biological pathways.

##### 2.1.3.2 Candidate genes

Candidates for gene network construction in this study come from 3 aspects:

1. Genes related to narcolepsy from literature review, which were partly obtained by genome-wide association analysis and partly from case studies;
2. Potential susceptibility genes identified by the genome-wide association analysis conducted in this study;
3. The first 20 genes with the lowest *P* value identified by the case-control differential gene expression study using public databases.

##### 2.1.3.3 Pathway information arrangement

The KEGG database was used to retrieve candidate pathway information, analyze the gene pathway information, screen the pathway information for common pathways between genes, and eliminate the unrelated pathways.

##### 2.1.3.4 Construction of the gene network

Pathways in accordance with biological logic were kept, and pathways with approximate functions were eliminated. Then, a gene network with pathways as links and genes as nodes was established.

## 3 Results

### 3.1 Genome-wide association analysis

#### 3.1.1 The sample and clinical data

After a series of strict quality control measures were taken, 903 narcolepsy patients were included in the study. Table 1 shows their basic and clinical information.

**Table 1:**
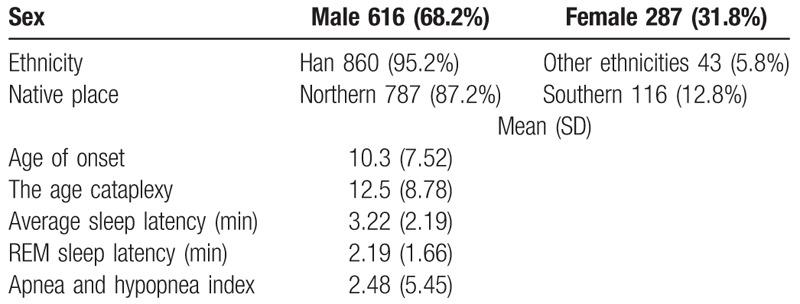
The basic and clinical information.

#### 3.1.2 The results of the principle component analysis

To avoid the impact of population stratification on the results, principle component analysis (PCA) was performed using genotype data from 237955 SNP loci. Most of the cases were concentrated in the same region as the control group, showing that the genetic background of the case group matched that of the control group. Some sample points deviate from the group, indicating that some population stratification existed. Fifteen sample points that exceed the average+ 6 SD (+0.114) were excluded. These 15 sample points belong to the control group. Therefore, 903 cases and 1982 controls were included in the final correlation analysis.

#### 3.1.3 Manhattan Diagram and Q-Q

**Figure 1:**
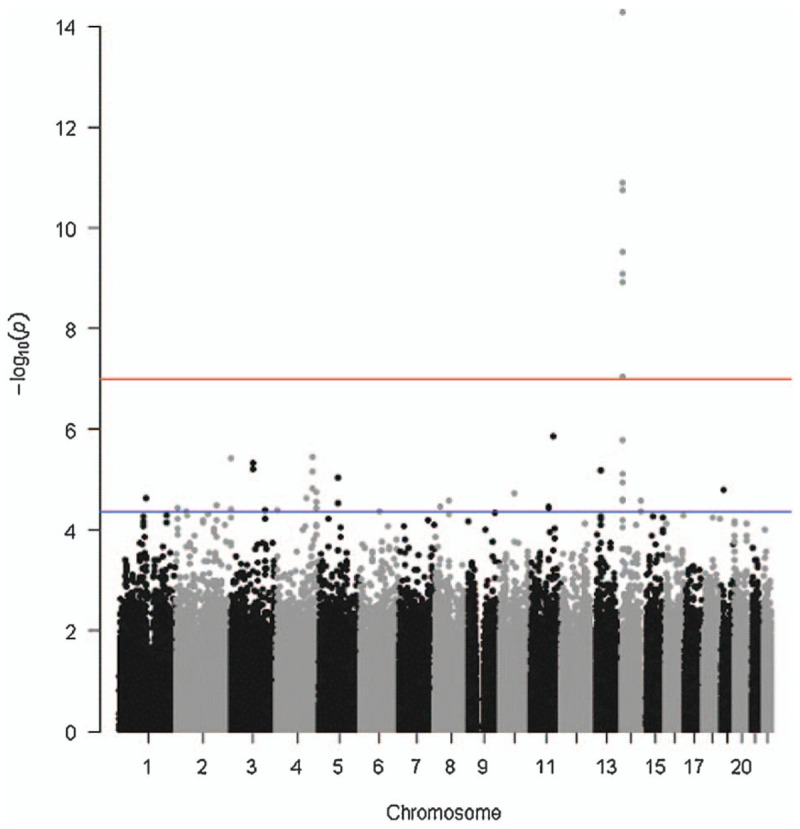
Manhattan plot of the results from the genome-wide association study.

In the case-control study, the genotype of SNP loci in non-HLA segments of autosomal chromosomes was correlated with the incidence of disease. Figure 1 is a Manhattan chart showing the overall results of genome-wide association analysis. The genetic variants of 7 SNPs exceeded the threshold for genome-wide significance in this GWAS after Bonferroni correction, and the genetic variants of more SNPs reached the significant threshold after false discovery rate (FDR) correction. The locus with lead SNPs within the TRA gene were among the top signals.

**Figure 2:**
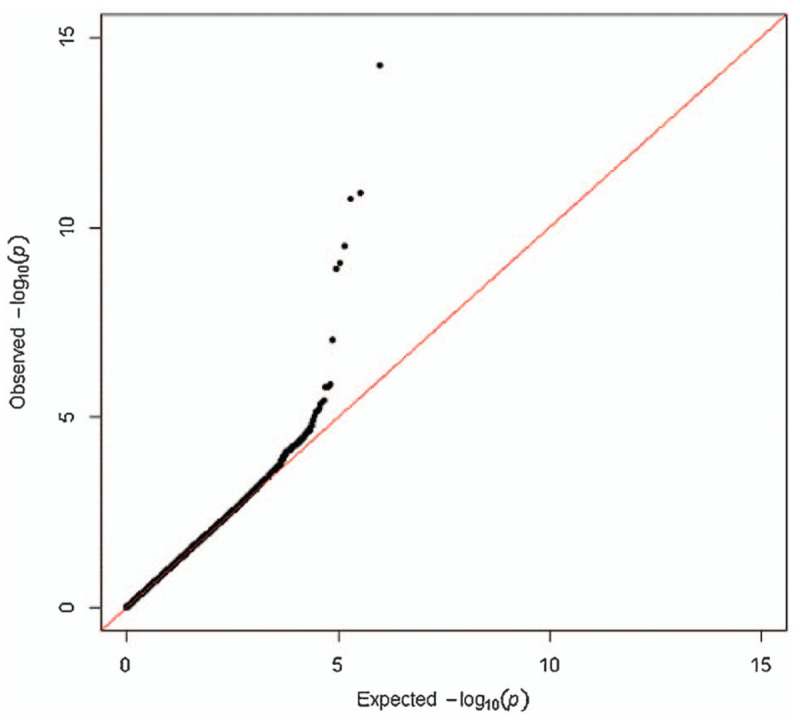
Quantile-Quantile plot.

Figure 2 shows the quantile-quantile plot (Q-Q plot) based on the *P* value of each SNP locus in the association analysis. The Q-Q plot is mainly used to show whether the observed values are significantly different from the predicted values. If the SNP loci are not related to disease, the *P* value obtained by analysis should accord with normal distribution, so the theoretical *P* value can be calculated. In the Q-Q plot, since most of the loci are not related to disease, the actual *P* value obtained is basically consistent with the theoretical *P* value, so most of the points in the plot are near the 45° diagonal line. Only a few of the data points are significantly related to the disease, so the actual *P* value obtained is larger than the theoretical *P* value. In addition, the genomic inflation factor (GIF) can be calculated according to the *P* value of the correlation analysis. If the λ value is too large, the population stratification value is too large in the experimental sample, which has a great impact on the experimental results. In genome-wide association analysis, it is generally believed that when 1 < λ<1.05, the population structure is relatively small and within acceptable range. In this study, the genome dilator λ value was 1.024. The Q-Q plot and λ value showed that mixed factors such as population stratification were well controlled in the association analysis.

The plot displays 903 individuals and 1982 controls. Single-nucleotide polymorphisms in black are in linkage disequilibrium with the index single-nucleotide polymorphisms and have a *P* value of less than .001. The abscissa represents the SNP loci on chromosome 1–22 in this study, and the longitudinal coordinate is –log 10(p). P is the *P* value of each SNP locus obtained from the correlation analysis. Larger values for -1og 10(p) indicate a greater the correlation between the SNP locus and the disease. The red line in the graph indicates the significant *P* value threshold of 1^∗^10–7 after Bonferonni correction, and the blue line indicates the significant *P* value threshold of 4.2^∗^10–5 after FDR correction.

After FDR correction and excluding the significant *TRA* locus on chromosome 14, the information for the remaining 25 SNP loci is shown in Table 2. Some loci are located in non-coding regions. Due to the difficulty of determining which genes are associated with these loci according to the number of existing loci, this was not analyzed in the follow-up study. Located within the gene, the functions *PPP2R2C, GPM6A, EPHX2, NKAIN3*, *ZNF365, TENM4,* and *PR2Y11* are related to narcolepsy. As these genes are potentially associated with narcolepsy, they will be incorporated into subsequent network construction.

**Table 2:**
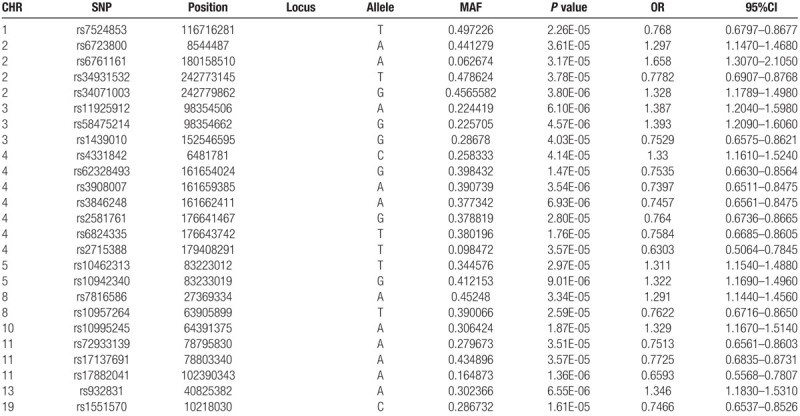
Significant SNPs after FDR correction.

### 3.2 Differential gene expression analysis

**Figure 3:**
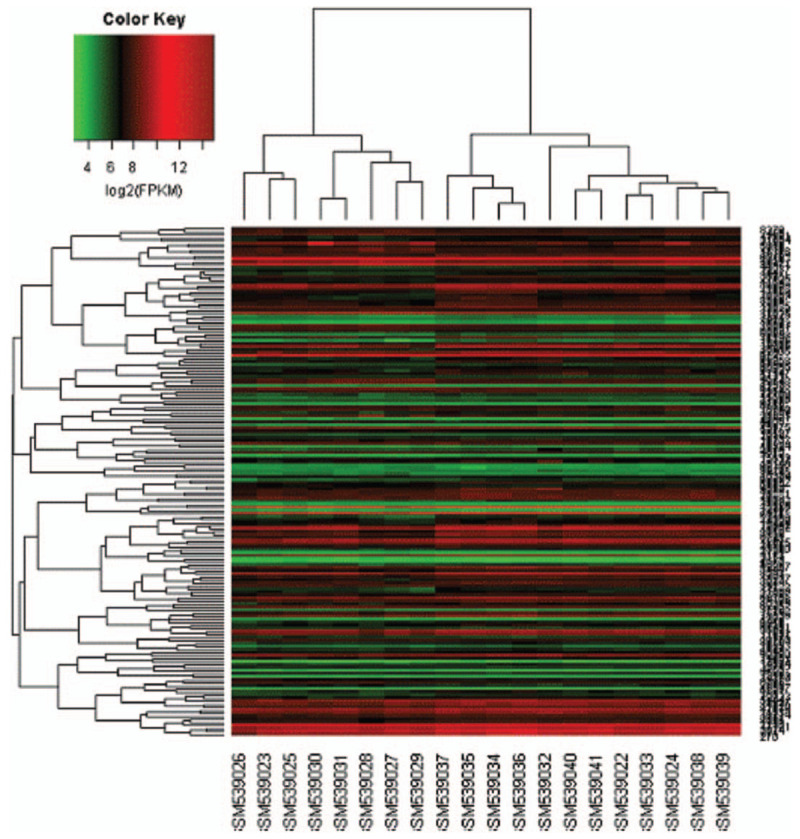
The thermal map of significant expression gene.

There were 168 genes in the differential gene expression analysis (*P* < .01). Thermal maps were drawn using these expression quantities (Fig. 3). The differences in gene expression between the control group and the case group are not noticeable in the figure. The most significant top 20 genes were selected for subsequent gene network construction. The selected gene information is shown in Table 3.

**Table 3:**
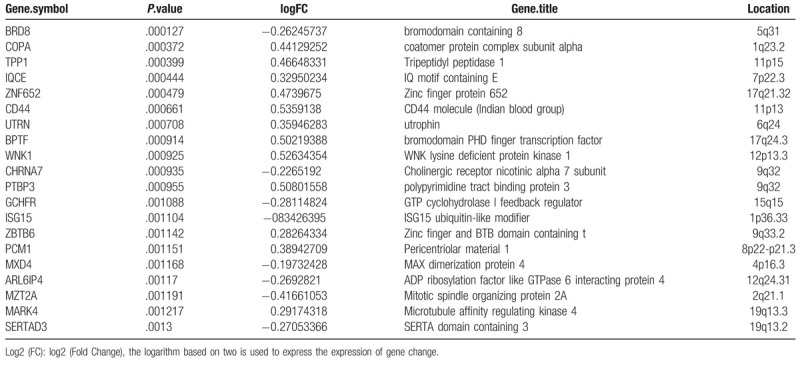
The information of the selected gene.

### 3.3 Construction of gene network

#### 3.3.1 Pathway information collection

The candidates for gene network construction in this study come from 3 aspects:

1. Thirty two genes related to narcolepsy were sorted out in the literature review stage;
2. Eight potential susceptible genes were identified by genome-wide association analysis in this study;
3. Differential gene expression was carried out using a public database.

The top 20 most prominent genes were selected. A total of 60 genes were identified and included in the subsequent analysis, which is summarized in Table 4.

**Table 4:**
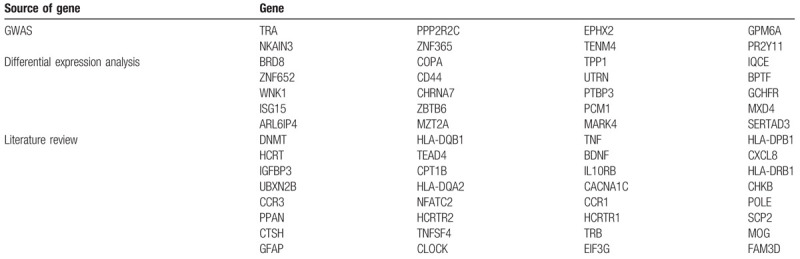
Summary of genes and sources.

Querying the KEGG database revealed that 33 of the 60 genes contain the pathway information. The results are shown in Table 5. Among them, the *HLA-DQB1, HLA-DRB1, HLA-DPB1,* and *HLA-DQA2* genes co-encode MHC class II molecules, so their pathway information was merged. After merging, the pathways involved in these 30 genes were sorted out to find the pathways in which they participated. A total of 28 genes were found to potentially connect with other genes through the pathways involved. The remaining two genes, *EIF3G* and *CHK*, were considered as isolated genes for the time being, and their pathways were not studied in the following steps.

**Table 5:**
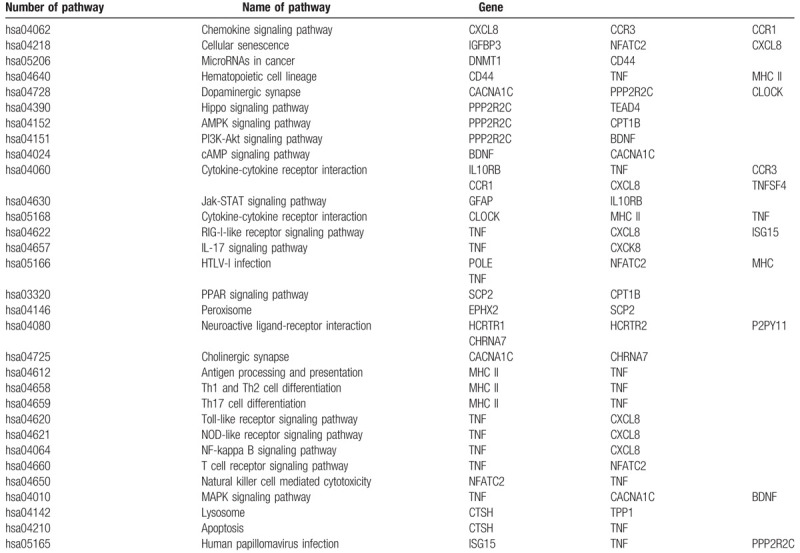
The path information in gene networks.

#### 3.3.2 Construction of Gene network

**Figure 4:**
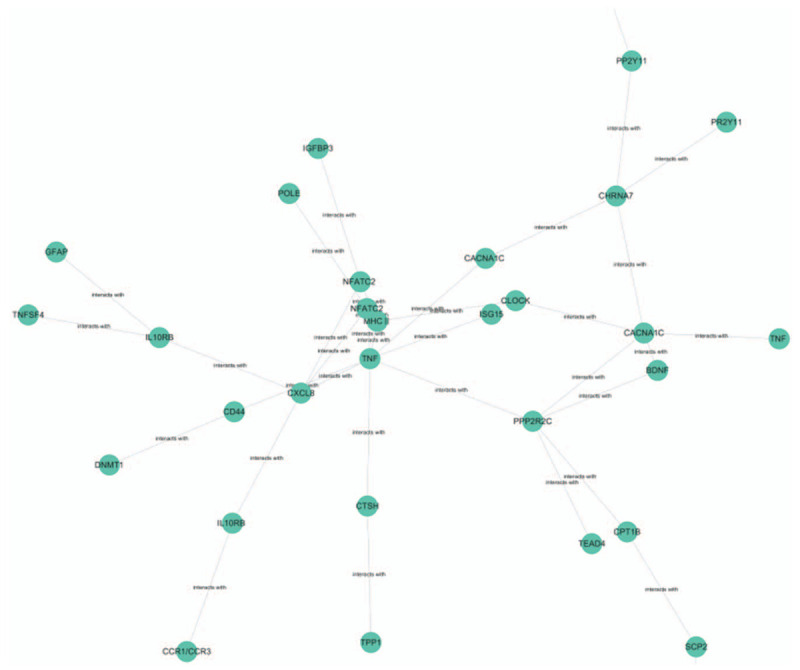
The gene network.

Of the pathways in which the genes involved were sorted out, only the biologically meaningful pathways were kept. Then, a network of 28 genes and 32 pathways was constructed. The gene network is shown in Figure 4.

## 4 Discussion

### 4.1 Analysis of GWAS results

In this study, a GWAS was conducted on 2885 case-control samples. The healthy controls were university staff and students, which may not reflect the normal control population. In order to avoid the potential impact of population stratification on the results, a principle component analysis was used to verify that the genetic background of the case group matched that of the control group. There may be some false positive loci in the results. In subsequent studies, it would be beneficial to use other datasets or carry out experimental analysis to test the loci, so as to increase the reliability of the results. Some SNPs located in non-gene-coding regions were also found in GWAS research. It is difficult to identify the functions of these regulatory genes. In the follow-up study, we may consider using eQTL (expression Quantitative Trait Loci) analysis, introducing gene expression data from different tissues, and establishing the relationship between gene expression and SNP loci in order to learn which genes are regulated by the SNPs of the non-gene-coding regions found in this study.

### 4.2 Biological significance of the gene network map

The gene network map is based on MHC II (major histocompatibility complex II) and *TNF* genes. The pathways involved include immune-related viral infections, antigen-presentation-related viral infections, antigen-presentation-related T cell signaling pathways, lectin receptor cytokine interaction, and a series of other pathways. Pathway types include dopamine synapses, cholinergic synapses, and ligand-receptor interactions with neurotransmitters associated with the nervous system. Existing evidence shows that narcolepsy type I may be caused by an autoimmune response in which hypothalamic secretin neurons are attacked. However, no antibodies or T cells corresponding to this process have been found, and the presence of the blood-brain barrier prevents antibodies from easily contacting the brain, so the specifics of pathogenesis are not clear. In this study, the genes associated with narcolepsy type I were linked by gene pathways, and the gene network map was drawn accordingly. Linkage between the nervous and immune systems was highlighted, which brought to light potential pathogenesis pathways for narcolepsy and provided a reference for studying its mechanisms and treatment methods.

### 4.3 Limitations

This study encountered several limitations. Only gene expression data from European patients were included, since the genetic variation underlying narcolepsy may differ across different ethnicities. Future studies with Asian narcoleptic patients will be possible when the necessary data are available. Another limitation of the study was that the healthy controls were university staff and students, rather than a completely random sample, In order to avoid bias, principal component analysis was carried out in this study. The results showed that the genetic background of the case group and the control group matched. Additionally, there may be some false positive loci in the results. In the follow-up study, different datasets may be used or experimental analysis to test the loci may be carried out, so as to increase the reliability of the results.

## Acknowledgments

We thank Dr Fang Han, Prof. Yiqun Wu and Prof. Dafang Chen for their valuable contributions.

## Author contributions

Study design: Jun Zhang, Hui Ouyang, Study performance: Hui Ouyang, Shiying Wang and Jun Zhang. Data analysis and interpretation: Shiying Wang and Qiwen Zheng. Paper writing: Hui Ouyang. All authors approved the final version of the paper.
